# Reducing blood pressure variability–results from a single-arm proof of concept prospective trial

**DOI:** 10.1038/s41598-025-14968-z

**Published:** 2025-08-12

**Authors:** Eyal Shemesh, Deborah Reynolds, Jasleen Sidhu, Sarah Duncan-Park, Rebecca A. Tejiram, Beth A. Davison, Koji Takagi, Chris Edwards, David Rubinstein, Rachel A. Annunziato, Gad Cotter

**Affiliations:** 1 Icahn School of Medicine at Mount Sinai, 1 Gustave L Levy Place, New York, NY 10029 USA; 2 Mindich Child Health and Development Institute, Precision Behavioral Medicine Program, Icahn School of Medicine at Mount Sinai, New York, NY 10029 USA; 3 NYC Health + Hospitals/Elmhurst, Queens, New York, NY 11373 USA; 4 Department of Psychology, Fordham University, Bronx, New York, NY 10458 USA; 5 Tulane University School of Medicine, New Orleans, LA 70112 USA; 6 Momentum Research, 1426 East NC Highway 54, Suite B, Durham, NC 27713 USA

**Keywords:** Blood pressure, Cardiovascular, Variability, Cardiology, Health care, Medical research, Risk factors

## Abstract

Increased variability in systolic blood pressure, expressed as the coefficient of variation (BPCoV), is associated with poor cardiovascular outcomes. Variability could be due to episodic non-adherence to medical recommendations in some patients. Reports of targeted attempts to mitigate such variation are lacking. A behavioral intervention targeted at patients with initially high BPCoV may decrease variability. In this single-site, single arm prospective proof-of-concept trial, an electronic health record review identified patients with excessive variability (BPCov>10%). Enrolled patients received a blood pressure monitor and a remotely delivered behavioral intervention for 3 months. The primary outcome was mean blood pressure variability before versus after the intervention. Of 551 initially screened patients, 107 (19.4%) met the BPCoV criteria, and 25 consented (6 females and 19 males, mean age 64.24 years). Average BPCoV for the 6 months pre-enrollment was 12.96 (*SD*=2.11) compared to 7.02 (*SD*=3.54) during intervention (*p*<0.001). Other variability metrics also improved. Sensitivity analyses (different timeframes, using measurements obtained in the clinic vs. home monitor) all showed significant improvement. This proof-of-concept trial suggests that patients with high systolic blood pressure variability can successfully engage in a remotely delivered behavioral intervention, and that such an intervention can reduce such variability.

**Trial Registration:** NCT05814562, ClinicalTrials.gov.

## Introduction

Systolic Blood Pressure Variability (BPV) is an independent risk factor for cardiovascular events, dementia, stroke, renal function decline, and mortality^1–4^. BPV represents a potentially useful marker that could “flag” at-risk patients, even if their blood pressure (BP) seems to be within normal limits and well controlled. But to date, there have been very few attempts to investigate interventions that may reduce such variability. Many factors contribute to higher BPV such as older age, female sex, obesity, alcohol consumption, smoking, stress, presence of end organ hypertensive (HTN) damage such as cardiac, kidney disease or atherosclerosis as well as time, day and season^5–9^. However, the only interventions shown to reduce BPV were therapies that reduce BP, especially calcium channel blockers^10^. In addition to biological causes, high BPV may be related to inconsistent medication-taking practices or non-adherence to other medical recommendations, resulting in an unstable BP over time. Non-adherence to medical recommendations, particularly to prescribed medications, is an important predictor of poor cardiovascular outcomes, and BPV might be an indicator, as well as a result, of such non-adherence.

The present proof-of-concept prospective feasibility pilot intervention trial (CP&R – Cardiovascular Precision medicine & Remote intervention) was designed to explore whether a tailored behavioral intervention, using the principles of “hovering” and addressing posttraumatic avoidance of medication-taking (when it is present), might be associated with a decrease in BPV in patients with an initially high BPV.

## Methods

### Participants

A full description of the methodology of this single-arm, proof of concept trial which compares pre-intervention vs. during intervention results in the same patients has been published elsewhere^11^.

In brief, adult participants with documented hypertension and hypercholesterolemia were eligible if a review of the electronic health record (EHR) found a high (>10%) systolic BP coefficient of variation (BPCoV – the standard deviation of at least three BP readings divided by the mean)^12^. Those meeting criteria were approached during a standard care clinic appointment.

Patients with a current psychiatric or developmental disorder that prevented them from understanding the protocol or engaging in the intervention (e.g., autistic disorder, psychosis) were excluded. Other exclusion criteria included suffering from a medical disorder that makes control of BP especially challenging or unlikely (e.g., end stage renal disease, uncontrolled endocrine disorders); unstable BP or hyperlipidemia that may require change in therapy in the 3 months after enrollment; significant heart failure (New York Heart Association score > 2) or ejection fraction < 50%; recent thromboembolic events such as a myocardial infarction, stroke, acute coronary syndrome; transient ischemic attack in the 6 months prior to enrollment; arrhythmia requiring medical or device therapy within 6 months prior to enrollment; hospitalization in the last 6 months prior to enrollment (but hospitalization after enrollment did not lead to withdrawal).

Screening and enrollment were done in the cardiovascular clinic at the NYC Health + Hospitals facility at Elmhurst in the borough of Queens, New York City, which serves one of the most diverse neighborhoods in NYC. The availability of language interpretation services and multilingual staff makes it possible to cater to the diverse population, although the remote intervention was delivered in this pilot only to English- or Spanish-speaking patients. The hospital pharmacy provides affordable medications to patients without insurance, ensuring that cost is not a barrier to standard care^13^.

### Study design

Chart reviews for screening were approved as a HIPAA (Health Insurance Portability and Accountability Act) waiver. Informed Consent was obtained from all patients at enrollment. The study was approved by Mount Sinai’s Program for the Protection of Human Subjects and by Elmhurst’s Research Committee and registered at clinicaltrials.gov (NCT05814562) on 4/3/2023. Research was performed in accordance with the Declaration of Helsinki and all methods were performed in accordance with the relevant guidelines and regulations. Participants could withdraw from the study if they declined to continue having their data collected from the medical charts. Patients who requested that we not call them anymore but who allowed continued data collection were not withdrawn from the study. If participants withdrew prior to the completion of follow up, pre-withdrawal data were used for the primary intent-to-treat analysis unless prohibited by the patient.

### Remote Intervention

All enrolled patients were given an automatic home BP monitor (same brand given to all patients). During the three months after enrollment, patients were administered the remote intervention weekly during the first month, and biweekly during the second and third months. The protocol allowed variations from this schedule based on patient preference, clinical imperatives, or PI decision. During each session, participants were asked to measure their BP, and this was recorded by the interventionist. Neither patients nor interventionists were masked to study aims.

The study utilized a tailored telemetric intervention to improve medication adherence. The telemetric intervention (offered via telephone or interactive internet chat applications such as Zoom ©, FaceTime ® or Skype ®) enabled experienced interventionists to address nonadherence from a remote location, using a structured, tailored approach that accommodates specific patient needs. Spanish-speaking patients were contacted and treated in their native language through an interpreter (via Pacific Interpreters ©). During the 8-session intervention, the interventionist reviewed the symptoms and risks associated with avoidance of medications due to illness-related distress, and supported the individual in considering and implementing remedies to such distress. In addition, adherence barriers were reviewed and addressed as needed, utilizing mainly problem-solving approaches, with help from the treatment team if necessary.

Caretakers or any individual identified as potentially supportive may have been included in the session at the interventionist’s discretion in accordance with patient preference. We offered no monetary incentives or reimbursements for participation.

If significant psychopathology, or any other substantial psychosocial risk, or any medical risk, was discovered during the intervention sessions, the participant was asked permission to share this information with the clinical team. With permission, the team was notified, and provided clinical evaluation and treatment. Intervention sessions were intended to last between 20-60 minutes. Audio of all encounters (web or phone) was recorded for a fidelity review. Fidelity to the essential intervention components (as defined in the manual) was verified by scoring a random sample of recorded sessions by an independent trained assessor who was not an interventionist in the study.

### Schedule of events

**Fig. 1:**
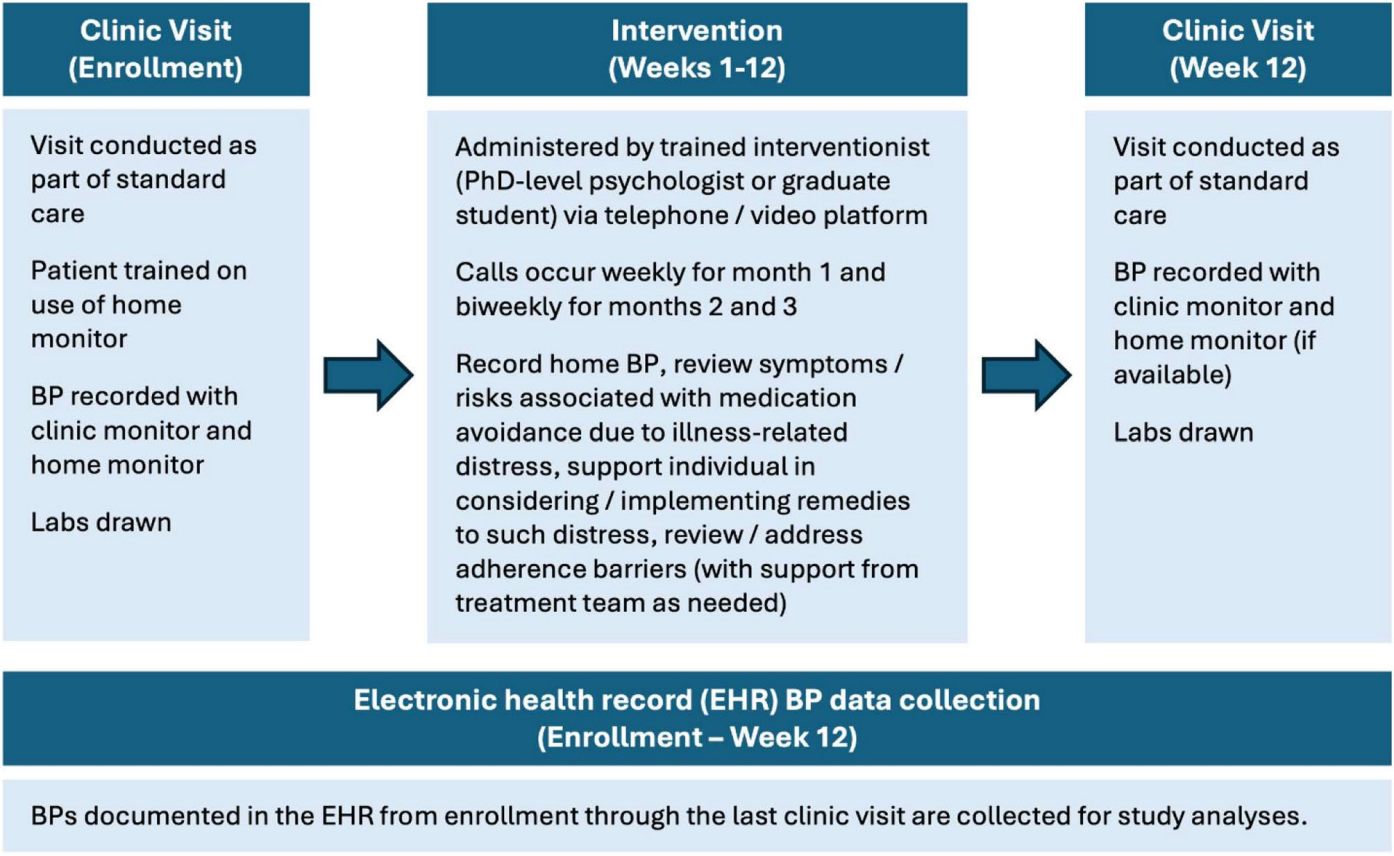
Schedule of events once patients were enrolled.

Table 1 and Figure 1 specify the schedule of assessments/events.

**Table 1:** Schedule of assessments.

Procedure	Visit 1*	Visit 2	Visit 3	Visit 4	Visit 5	Visit 6	Visit 7	Visit 8	Visit 9*
	Enrollment	Week 1	Week 2	Week 3	Week 4	Week 6	Week 8	Week 10	Week 12
Eligibility	X								
Informed Consent	X								
BP (Clinic)	X								X
Blood sample for cholesterol	X								X
BP (Home monitor)	X	X	X	X	X	X	X	X	X
Remote intervention		X	X	X	X	X	X	X	X
Adverse event monitoring	<------------------------------------------------X--------------------------------------------------->								

^*^Clinic visit.

#### Biological specimen collection and laboratory evaluations

A blood sample of approximately 5.0 mL (cc) was withdrawn at Visits 1 (enrollment) and 9 (Week 12) for measurement in the local laboratory, including total cholesterol, LDL, HDL, and triglycerides.

#### BP measurements

BP was manually measured by clinic nurses during clinic visits at enrollment, during the study (as needed), and at the end of the study, following clinical protocols. Participants were provided with a BP home monitor and trained in its use at enrollment. Patients were instructed to measure their BP using their home monitor at the beginning of each intervention call in the same arm after resting seated in a chair for 5 minutes. Patients were instructed to sit in a dining-room-type chair with feet flat on the floor with uncrossed legs and back straight, with the arm supported on a flat surface and the upper arm at about heart level. The result was verbally communicated to the study interventionist and recorded. The unit of analysis was mm/Hg.

### Outcome measures and hypotheses

We first aimed to assess implementation of the screening procedures and intervention as follows: 1) identify the proportion of clinic patients with a BPCoV >10% (“at-risk” patients) based on EHR review (hypothesis 1.1: Between 10- 20% of clinic patients will display “at risk” pattern of BPCoV >10%); 2) enroll a subset of these at-risk patients in the intervention study (hypothesis 1.2: At least 50% of those who are “high risk” and approached will agree to participate); and 3) engage these at-risk patients in at least three full remote intervention sessions over the study period (hypothesis 1.3: At least 50% of those who are recruited will participate in at least 3 remote intervention sessions).

We then sought to evaluate the impact of the intervention on patient outcomes using pre-post comparisons across the following three areas: 1) BPV (hypothesis 2.1: BPV will decrease in the 3 months since enrollment as compared with the 6 months pre-enrollment); 2) cholesterol levels (hypothesis 2.2: Cholesterol levels will decrease from enrollment visit to 3 months follow-up visit); and 3) BP (hypothesis 2.3: BP will decrease from enrollment visit to 3 months follow-up visit).

### Statistical analysis

Descriptive statistics were performed to summarize study data. Sample size (n=25 enrolled, to provide at least 20 analyzable subjects) was pre-determined to provide power to detect modest pre-post changes in continuous measures (80% power to detect a change of 0.58 standard deviations at the one-sided 0.05 significance level).

Paired samples t-tests were used to compare BP fluctuation and clinical endpoints before and after the intervention. Systolic BP readings are presented. For the primary analyses, systolic BP fluctuation was measured using BPSDV - the standard deviation of at least three BP measurements within a specified period (e.g., 6 months pre-intervention); BP fluctuation was also measured using the BPCoV method.Supplemental analyses were performed to consider the effect of analysis timeframe and BP measurement source (i.e., measured by a nurse in the clinic vs. by the home monitor) on BPV. Hypothesis testing was performed at the one-sided α=0.05 significance level. No adjustments for multiplicity were made. Analysis samples varied by specific aim and analysis. For example, the primary analysis comparing BP fluctuation before and after the intervention includes only participants with at least three readings in the six months pre-enrollment and at least three readings at weeks 2, 4, 8, and 12. An intent-to-treat paradigm with last observation carried forward was used to compute differences in blood pressure readings between pre-intervention to the end of the intervention.

## Results

### Screening results and characteristics of recruited sample

**Fig. 2:**
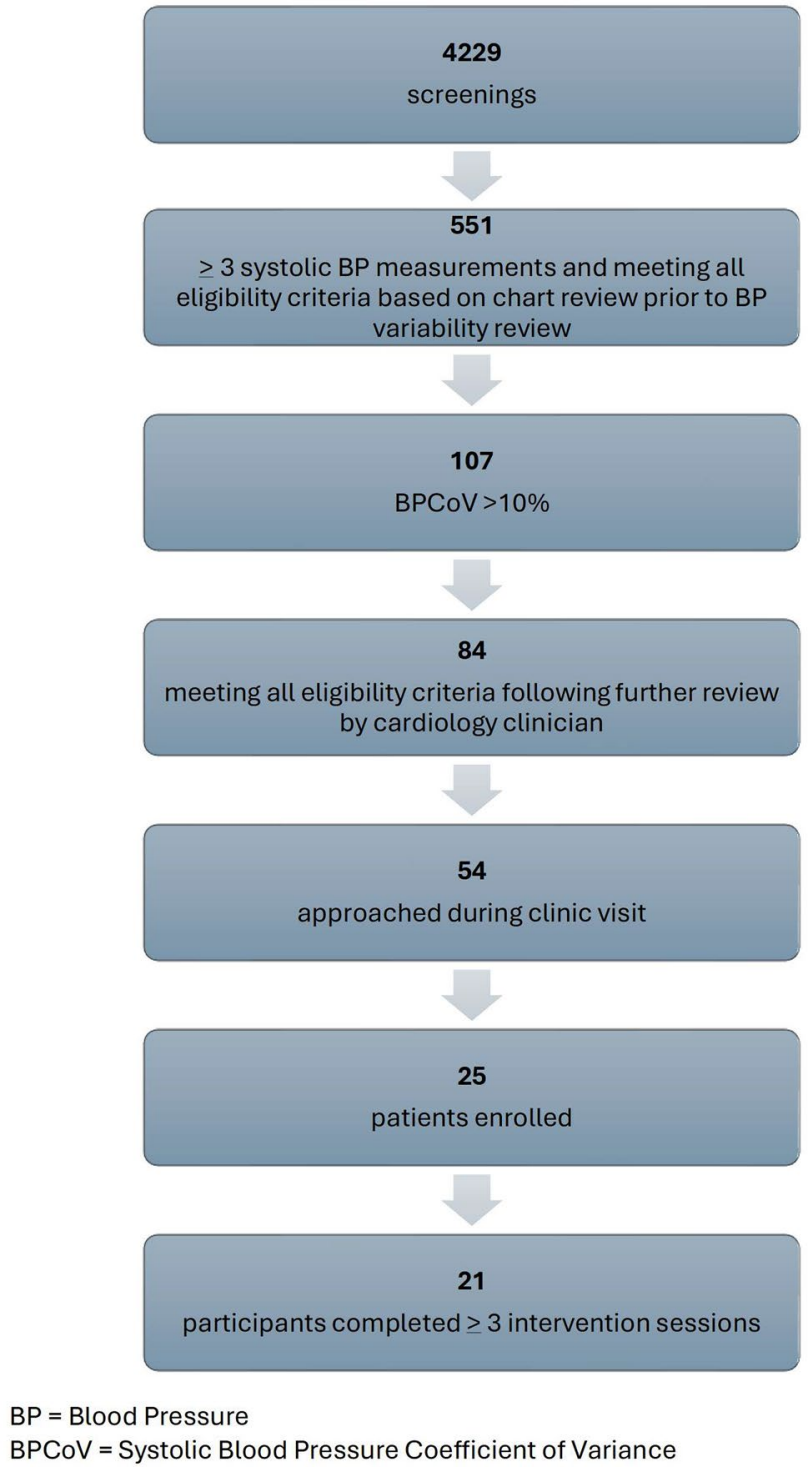
Recruitment diagram.

The study was approved on 6/17/2022 and closed on 2/24/2025. Of 4229 screenings, 551 patients met baseline (age, cardiovascular baseline parameters) eligibility criteria, and 19.4% of those (n=107, 90% CI=16.8%, 22.3%) also met the BPCoV criteria (Figure 2). We were able to approach 64% of those who met the full eligibility criteria, and 46.3% (n=25, 90% CI: 35.5%, 57.4%) of those approached agreed to participate. Of the 25 enrolled patients, 84.0% (n=21, 90% CI: 69.4%, 93.1%) fully participated in at least 3 sessions (i.e., completed at least 3 sessions including performing at least 3 home BP readings). Baseline demographic characteristics of the recruited sample are available in Table 2. and demonstrate a preponderance of male participants with a racial and ethnic mix that is characteristic of the Elmhurst community.

**Table 2:** Baseline characteristics of study participants.

**Variable**	**N = 25**
Age (years) Mean (SD)	64.24 (8.52)
Sex assigned at birth, N (%)	
Female	6 (24.0)
Male	19 (76.0)
Intersex	0 (0)
Other	0 (0)
Not reported	0 (0)
Gender, N (%)	
Woman	6 (24.0)
Man	19 (76.0)
Transgender/trans man	0 (0)
Transgender/trans woman	0 (0)
Non-binary	0 (0)
Other	0 (0)
Not reported	0 (0)
Race, N (%)	
American Indian or Alaska Native	0 (0)
Asian	7 (28.0)
Black or African American	3 (12.0)
Native Hawaiian or Pacific Islander	1 (4.0)
White	1 (4.0)
Not reported	13 (52.0)
Ethnicity, N (%)	
Hispanic or Latino	12 (48.0)
Not Hispanic or Latino	13 (52.0)
Not reported	0 (0)
Marital status, N (%)	
Divorced	3 (12.0)
Married	12 (48.0)
Single/Never married	8 (32.0)
Separated	0 (0)
Widowed	1 (4.0)
Other	1 (4.0)
Language, N (%)	
English	16 (64.0)

### Differences between the clinic-obtained vs. home-monitored BP (A manipulation analysis)

In the enrollment visit, where both the home monitor’s reading and the clinic’s reading were recorded contemporaneously, there was a consistent difference between the home monitoring reading and the clinic measurement. Systolic BP, mean (SD) measured by the home monitor was 127.14 mmHg (15.92), whereas the clinic value was 124.36 (10.67), denoting a higher reading by the home monitor by an average of about 3mmHg, reflecting a systematic effect of the method of blood pressure measurement.

### Primary analyses

#### Blood pressure variability (expressed either as BPSDV or BPCoV)

**Fig. 3:**
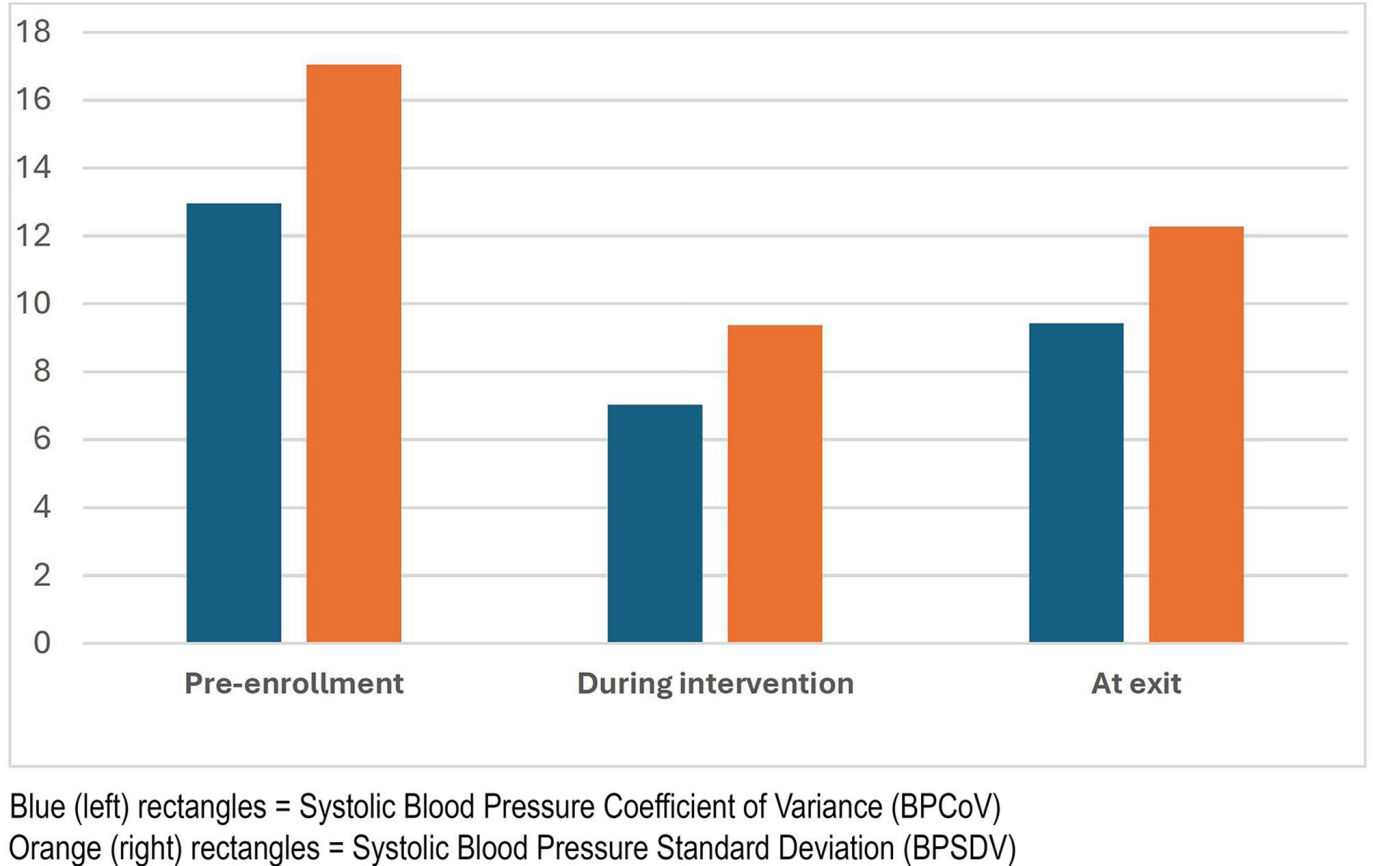
Decrease in variability during intervention and at exit.

Sixteen participants had at least 3 BP readings 6-months pre-enrollment and 3 BP readings at study weeks 2, 4, 8, and 12. If no measure was available at a particular week, the closest measure in time was used. We analyzed the data using the two prevalent ways to measure variability: either as a standard deviation of a set of values (BPSDV) for each participant, or as the coefficient of variation (BPCoV) – the ratio of the SDV to the mean value for each participant. Average BPSDV pre-enrollment was 17.04 (*SD*=3.11) compared to 9.37 (*SD*=4.97) during the intervention (*p*<0.001). Average BPCoV for the 6 months pre-enrollment was 12.96 (*SD*=2.11) compared to 7.02 (*SD*=3.54) during intervention (*p*<0.001). Figure 3 shows the differences in BPCoV and BPSDV as measured pre-intervention, during intervention (at the end of 3 months) and at study exit (at the end of 6 months). The decrease in variability was most pronounced during the intervention. The differences between the timeframes after enrollment were not statistically significant.

#### Change in cholesterol and triglyceride levels

All participants (n=25) had lab results available at enrollment and at the end of the study. The average difference in participants’ total cholesterol, LDL, and HDL at enrollment compared to the end of the study was not statistically significant (total cholesterol mean difference:0.60 mg/dL, *SD*=24.91, *p*=0.45; LDL mean difference: −4.54 mg/dL, *SD*=22.90, *p*=0.17; HDL mean difference: 1.40 mg/dL, *SD*=6.79, *p*=0.16). Participants’ average triglycerides were significantly lower at the end of the study (125.80 mg/dL, *SD*=66.16) compared to at enrollment (148.24 mg/dL, *SD*=77.35, *p*=0.02).

#### Change in BP

Participants’ (n=25) average clinic-measured systolic BP was not significantly different at the time of enrollment (124.32, *SD=*10.07) compared to the end of the study (126.04, *SD*=9.50, *p*=0.23).

### Sensitivity analyses

Supplemental analyses were performed to consider the effect of post-enrollment timeframe (e.g., all readings 3 months post-enrollment instead of only readings at weeks 2, 4, 8, and 12) and BP measurement source (e.g., home monitor, EHR) on the BPV results. BPV remained significantly lower post-enrollment compared to pre-enrollment regardless of the timeframe used in the analyses and whether the analyses included only home monitor or home monitor plus EHR BP readings (Table S1).

## Discussion

Our pilot investigation confirmed that at-risk patients with high BPV^14^ can be identified via EHR information, and that once identified, those patients can be engaged in a remote behavioral intervention. During the intervention, average BPV significantly decreased, but average systolic blood pressure remained the same. Therefore, the decrease in variability was not due to a more general decrease in BP, but possibly due to more consistent adherence to medical recommendations. Also of note, patients’ initial (enrollment visit) blood pressure in this study was not elevated, suggesting that we were able to focus on patients who wouldn’t have necessarily been considered “at risk” unless the variability metric was screened for.

Almost all previous studies that have shown BPV reduction were sub-analyses of studies in which patients with hypertension were treated. In those studies, BP readings were decreased in parallel to BPV^10^. To the best of our knowledge, only one study has targeted BPV specifically^15^. In this study, the investigators did not see improvement in BPV after treatment to reduce weight and reduce salt intake, but that study did not require a high BPV at enrollment. The present study is the first, to our knowledge, to pre-identify patients with high BPV, using a “precision medicine” paradigm to target the patients with the highest risk. In addition to pre-selecting “high-risk” patients based on available EHR data, we aimed to reduce patient burden by focusing our enrollment efforts in a clinic setting, and employing a completely remote intervention. This potentially helped in recruitment and follow-up of patients who are otherwise hard to engage. The fact that we were able to identify and enroll this very selective group of patients and engage them in a 3-month long intervention is important on the way to design a more rigorous investigation.

Our intervention specifically targeted medication-taking behavior, with an additional focus on stress-related medication avoidance (the phenomenon in which taking the medicine reminds patients that they are ill, they find such reminders stressful, and therefore stop taking the medicine). We previously noted that stress-related avoidance is indeed one reason for non-adherence to medications and that such distress can be ameliorated (in a study conducted in the same clinic^16^). But our intervention did not include any form of an established psychological treatment (it cannot be considered a form of cognitive behavioral therapy for example). Addressing mental health disorders was completely outside the scope of this investigation.

Remote interventions are considered at the forefront of adherence research, as they help engage patients who are particularly challenging to engage. A team-based remote approach was shown to improve the uptake of guideline-directed therapy in patients with type II diabetes^17^, and a recent review and meta-analysis of remote interventions concluded that they reduce BP and cholesterol concentration in patients after a stroke, improve adherence, and result in high patient satisfaction ratings^18^.

Some limitations of this work must be acknowledged. The intervention was short by necessity (a limited investigation) – a longer intervention might have resulted in greater benefits. Of 84 patients who met inclusion criteria following chart and clinician review, we were able to approach 54 due to the study’s limited timeframe (we only approached patients who came to clinic, and adherence to appointment schedules may be particularly challenging for our target population), 25 enrolled, and 21 finished our pre-defined session minimum. Some selection bias is likely, but given this particularly challenging population, we expected even more patients to not show up to their clinic appointments or decline participation. The fact that a large proportion of enrolled patients did participate in our pre-set minimum number of sessions suggests that in general, we were able to both identify and engage this hard-to-engage population. The most non-adherent patients, those who do not attend their medical appointments, have likely not been enrolled; this population might need a more intensive intervention paradigm, for example home visits. Our results therefore only pertain to patients who are non-adherent enough so that their blood pressure fluctuates but are not completely lost to follow-up. We found that home monitoring resulted in slightly elevated readings as compared to the readings in the clinic, though the white coat effect is generally assumed to increase measured blood pressure in clinics, not at home. Our home monitor was used during a session with our interventionist, not while alone, and recorded then. Thus, the white coat effect in this study is expected to affect both clinic and home measurements (as compared with a situation in which patients take their home blood pressure alone and without the presence of a clinician). We therefore believe that the differences observed are due to a slight calibration difference between machines. As in any uncontrolled investigation, some of our observed results might be partly attributed to “regression to the mean”. The fact that both BPCoV readings (which were used as a selection criterion) as well as BPSDV readings (which were not our selection criterion) improved, somewhat mitigates that concern. Only a randomized controlled trial can confirm an intervention’s efficacy. Blood pressure did not decrease, only variability did, establishing that BPV reduction was not due to a more general drop in BP. This is of importance given that the patients had well controlled BP at baseline, further suggesting that if the variability marker was not used, those patients would not have been identified by the clinic as being “at risk”. It is not clear which specific element in the intervention was responsible for the BPV improvement, and whether simply increased attention to patients and frequent monitoring might improve BPV (without the need for a more elaborate intervention). Even if our results were due primarily to attention and monitoring, and not due to any specific elements of the remote intervention, this would not affect the conclusion that patients with increased variability can be successfully targeted for a remote intervention aiming to improve adherence to medical recommendations, and that such an intervention might mitigate cardiovascular risk.

In conclusion, this pilot investigation found that it is possible to engage patients with a high-level BPV in a remote behavioral intervention and that such engagement is associated with a substantial decrease in such variability. Our results strongly suggest that a high level of variability can be a result of a behavioral risk, rather than being a purely biological construct (because such putative biological risks are not likely to be mitigated so quickly by a behavioral intervention). A controlled study of longer duration (given our observation that the intervention effect may not have been fully sustained after three months) can further elucidate whether our EHR-based risk stratification could result in a “precision medicine” patient-centered, cost-effective approach to identify and successfully intervene with a highly selective group of at-risk patients.

## Supplementary Information

Supplementary Information.

## Data Availability

Data are not available for general use as per our IRB policies, but will be available upon request from the journal’s editors.

## Abbreviations

BPCoV: Systolic Blood Pressure Coefficient of Variation
BPV: Blood Pressure Variability
BPSDV: Systolic Blood Pressure Variability Index, expressed as standard deviation of a series of measurements
CP&R: Cardiovascular Precision medicine & Remote intervention
EHR: Electronic Health Record
