# Supplementary material for: Reducing blood pressure variability–results from a single-arm proof of concept prospective trial
Source: Sci Rep. 2025 Aug 12;15:29449. doi: 10.1038/s41598-025-14968-z (PMC12340024; doi:10.1038/s41598-025-14968-z)
Supplement: Supplementary file 1 — Supplementary Information. [file 41598_2025_14968_MOESM1_ESM.docx]

**Supplemental Table**

**eTable 1. BP fluctuation using varied timeframes and measurement sources**

| **Time period** | **BP source** | **N** | **Mean (SD)** | **p-value** |
| --- | --- | --- | --- | --- |
| **Pre-enrollment BP fluctuation vs. fluctuation up to 3 months post-enrollment** | | | | |
| BPSDV 6 months pre-enrollment | EHR / clinic | 17 | 16.88 (3.09) | <.001 |
| BPSDV 3 months post-enrollment | HM |  | 9.43 (3.61) |  |
|  |  |  |  |  |
| BPCoV 6 months pre-enrollment | EHR / clinic | 17 | 12.92 (2.05) | <.001 |
| BPCoV 3 months post-enrollment | HM |  | 7.21 (2.65) |  |
|  |  |  |  |  |
| BPSDV 6 months pre-enrollment | EHR / clinic | 19 | 16.42 (3.71) | .01 |
| BPSDV 3 months post-enrollment | HM / EHR |  | 12.27 (5.68) |  |
|  |  |  |  |  |
| BPCoV 6 months pre-enrollment | EHR / clinic | 19 | 12.56 (2.65) | .01 |
| BPCoV 3 months post-enrollment | HM / EHR |  | 9.30 (4.10) |  |
|  |  |  |  |  |
| **Pre-enrollment BP fluctuation vs. fluctuation up to study exit** | | | | |
| BPSDV 6 months pre-enrollment | EHR / clinic | 17 | 16.88 (3.09) | <.001 |
| BPSDV all post-enrollment | HM |  | 9.21 (3.47) |  |
|  |  |  |  |  |
| BPCoV 6 months pre-enrollment | EHR / clinic | 17 | 12.92 (2.05) | <.001 |
| BPCoV all post-enrollment | HM |  | 7.05 (2.55) |  |
|  |  |  |  |  |
| BPSDV 6 months pre-enrollment | EHR / clinic | 20 | 15.78 (4.62) | .02 |
| BPSDV all post-enrollment | HM / EHR |  | 12.27 (5.16) |  |
|  |  |  |  |  |
| BPCoV 6 months pre-enrollment | EHR / clinic | 20 | 12.08 (3.36) | .02 |
| BPCoV all post-enrollment | HM / EHR |  | 9.42 (3.83) |  |

BP= Blood Pressure

BPSDV = Systolic Blood Pressure Standard Deviation

BPCoV = Systolic Blood Pressure Coefficient of Variance

HM – home monitor
